# Distribution of ischemic infarction and stenosis of intra- and extracranial arteries in young Chinese patients with ischemic stroke

**DOI:** 10.1186/s12872-015-0147-5

**Published:** 2015-11-23

**Authors:** Rajeev Ojha, Dongya Huang, Hedi An, Rong Liu, Cui Du, Nan Shen, Zhilan Tu, Ying Li

**Affiliations:** Department of Neurology, East Hospital Affiliated to Tongji University School of Medicine, Shanghai, 200120 China

**Keywords:** Young, Stroke, Chinese, Risk factors, Carotid stenosis, Multiple infractions

## Abstract

**Background:**

The distribution of cerebral ischemic infarction and stenosis in ischemic stroke may vary with age-group, race and gender. This study was conducted to understand the risk factors and characteristics of cerebral infarction and stenosis of vessels in young Chinese patients with ischemic stroke.

**Methods:**

This was a retrospective study, from January 2007 to July 2012, of 123 patients ≤50 years diagnosed with acute ischemic stroke. Patient characteristics were compared according to sex (98 males and 25 females) and age group (51 patients were ≤45 years and 72 patients were 46–50 years). Characteristics of acute ischemic infarction were studied by diffusion weighted imaging. Stenosis of intra- and extracranial arteries was diagnosed by duplex sonography, head magnetic resonance angiography (MRA) or cervical MRA.

**Results:**

Common risk factors were hypertension (72.4 %), dyslipidemia (55.3 %), smoking (54.4 %) and diabetes (33.3 %). Lacunar Infarction was most common in our patients (41.5 %). Partial anterior circulation infarction was predominant in females (52.0 vs 32.7 %; *P* = 0.073) and posterior circulation infarction in males (19.8 vs 4 %; *P* = 0.073). Multiple brain infarctions were found in 38 patients (30.9 %). Small artery atherosclerosis was found in 54 patients (43.9 %), with higher prevalence in patients of the 46–50 years age-group. Intracranial stenosis was more common than extracranial stenosis, and middle cerebral artery stenosis was most prevalent (27.3 %). Stenosis in the anterior circulation was more frequent than in the posterior circulation (*P* < 0.001).

**Conclusions:**

In these young patients, hypertension, smoking, dyslipidemia and diabetes were common risk factors. Intracranial stenosis was most common. The middle cerebral artery was highly vulnerable.

## Background

Although stroke in young adults is considered to be relatively rare, it is one of the leading causes of death and long-term disability worldwide. Its influence over a productive period of life in young stroke survivors might further cause psychological and social problems. Over the last decade, several studies have shown an increase in the number of young stroke patients. However, reports from developing and underdeveloped countries relating to young patients with stroke have been less frequent and were concerned with the higher frequency of undetermined causes of stroke [1–3].

Variations in the risk factors among populations from different countries may explain the differences in the incidence of stroke which ranges from 5–10 % [4–6]. Although several previous studies have been published on the risk factors, etiology and clinical outcome, only few studies have focused on cerebral vasculature of young stroke [7–11]. Such discrepancy might have been due to unavailability, high expense, invasiveness and concerns about the radiation hazard of the traditional neuroimaging tools utilized in determining the affected vasculature. In recent years, use of advanced non-invasive neuroimaging techniques such as diffusion weighted imaging (DWI) and magnetic resonance angiography (MRA) have helped us to better understand the vascular status and infarction lesions of young stroke patients [12, 13].

Different risk factors and etiologies influence the characteristics of the infarction lesion and intra- and extracranial stenosis [11, 14]. In developing countries, young people are largely unaware of the proper control of cardio- and cerebrovascular risk factors [14]. Previous stroke studies in China showed a higher prevalence of smoking and hypertension in young patients with ischemic stroke, but only a few studies have reported upon stroke etiology among Chinese stroke patients [2]. Although atherosclerosis and cardioembolism are the common etiological findings, it is very important to look for other determined causes of stroke in young patients. So, this study was conducted to analyze the risk factors and etiology, and understand the characteristics of cerebral infarctions and stenosis of intra- and extracranial vessels in young Chinese patients with ischemic stroke.

## Methods

All consecutive patients selected for the study, who had been admitted in department of neurology of East Hospital, Tongji University School of Medicine, Shanghai, China, between January 2007 and July 2012, were reviewed retrospectively. Patients were aged 15–50 years at stroke onset and had been newly diagnosed as having acute cerebral infarction [15]. The inclusion criteria for the study were: (1) acute ischemic stroke as confirmed by DWI; (2) age ≤ 50 years during onset; (3) patient underwent at least duplex sonography or head or cervical MRA. The exclusion criteria were: (1) cerebral or subarachnoid hemorrhage; (2) Transient ischemic attack. Out of 128 young ischemic strokes, 123 fulfilled the inclusion criteria. Five patients were excluded because there was no evidence of an infarction lesion in DWI. The demographic, clinical, investigation and radiological data of each patient were collected from the computer database of the hospital and was reviewed by a team of neurologists. We divided our patients according to sex (98 male and 25 female patients) and age group (51 patients were ≤45 and 72 patients were in range of 46–50 years) for further comparative study. The study protocol was approved by the Ethics Committee of East Hospital, affiliated to Tongji University School of Medicine.

A computed tomography (CT) scan (Siemens; USA) of the head was performed for all patients to rule out cerebral hemorrhage. All patients underwent a DWI scan of the head in a 1.5 T MRI Scanner (Philips; Holland) to confirm and locate the cerebral infarction. Extracranial arteries were examined to evaluate the vessels that correlated with the lesion site by duplex sonography (Micromaxx; Sonosite, USA) of 9 L4 linear array transducer with bandwidth of 4–9 MHz. 3D (TOF) time-of-flight. Head and cervical MRA were performed to study intra- and extracranial arteries respectively. Duplex sonography was performed in 96 (78 %), head MRA in 98 (80 %) and cervical MRA in 33 (27 %) patients. Duplex sonography or head/cervical MRA were used to define stenosis as narrowing of intra- or extracranial arteries by ≥ 50 % and were evaluated by 2 expert neuroradiologists who had more than 5 years of experiences in stroke diagnosis [16]. An arterial plaque was defined as a localized thickening > 1.2 mm in the artery, with or without flow disturbance [17].

All patients underwent routine blood tests, chest X-ray, electrocardiogram, lipid profile, coagulation profile, thyroid function test and transthoracic echocardiography. Patients were also screened for infectious diseases and vasculitis. The following risk factors for ischemic stroke were assessed: hypertension, smoking, hyperlipidemia, diabetes mellitus, previous myocardial infarction, transient ischemic attack or stroke, peripheral arterial diseases, atrial fibrillation, other cardiovascular diseases, history of migraine with aura, use of illicit drugs, pregnancy and use of oral contraceptive pills.

Hypertension was defined as a medical history of elevated blood pressure requiring treatment or persistently elevated blood pressure (systolic ≥ 140 mm Hg; diastolic ≥ 90 mm Hg). Diabetes mellitus was defined as a medical history of diabetes or patients with a fasting glucose ≥ 7.0 mmol/L (126 mg/dl) or a two-hour postprandial serum glucose ≥ 11.1 mmol/L (200 mg/dl). Dyslipidemia was defined as a medical history of hypercholesterolemia or a fasting serum low-density lipoprotein cholesterol ≥ 4.1 mmol/L (160 mg/dl), or a total cholesterol ≥ 6.3 mmol/L (240 mg/dl). Subjects were considered smokers if they had smoked for more than 5 years in the past, or reported current daily smoking of cigarettes, cigars or a pipe. Migraine stroke was defined according to the diagnostic criteria of International Headache Society [18].

Essen stroke risk score was calculated in all the patients [19]. The National Institutes of Health Stroke Scale (NIHSS), modified Rankin Scale (mRS) and Barthel Index were recorded to assess the clinical status and stroke severity during admission and discharge [20, 21]. The mRS scale was used for evaluating the degree of disability (Score range: 0–6), a low score signified that there were no neurological symptoms and a high score signified severe disability or death. On the other hand, a low score in the Barthel Index (Score range: 0–100) showed a weak performance of patient in terms of activities of daily living, and a high score signified a better performance.

Oxfordshire Community Stroke Project (OCSP) [22] classification was used to clinically classify the ischemic stroke into total anterior circulation infarction (TACI), partial anterior circulation infarction (PACI), lacunar infarction (LACI) and posterior circulation infarction (POCI). Trial of Org 10172 in Acute Stroke Treatment (TOAST) classification [23] was used to determine the etiology of ischemic stroke which was classified into cardioembolism, large artery atherosclerosis (LAA), small artery atherosclerosis (SAA), other determined etiology (ODE) and undetermined etiology (UDE). Multiple brain infarcts (MBI) were defined as more than 1 lesion present in single or more than one vascular territory.

SPSS for windows 14.0 was used for data storage and statistical analysis. Pearson Chi-Square test and Fisher exact test were performed to compare categorical variables whereas Student t test was used to compare continuous variables. A two-tailed *p*-value < 0.05 was considered statistically significant. A normality test was performed on the data and verified. Age, Essen stroke risk score, NIHSS, mRS and Barthel Index were the quantitative variables.

## Results

**Fig. 1:**
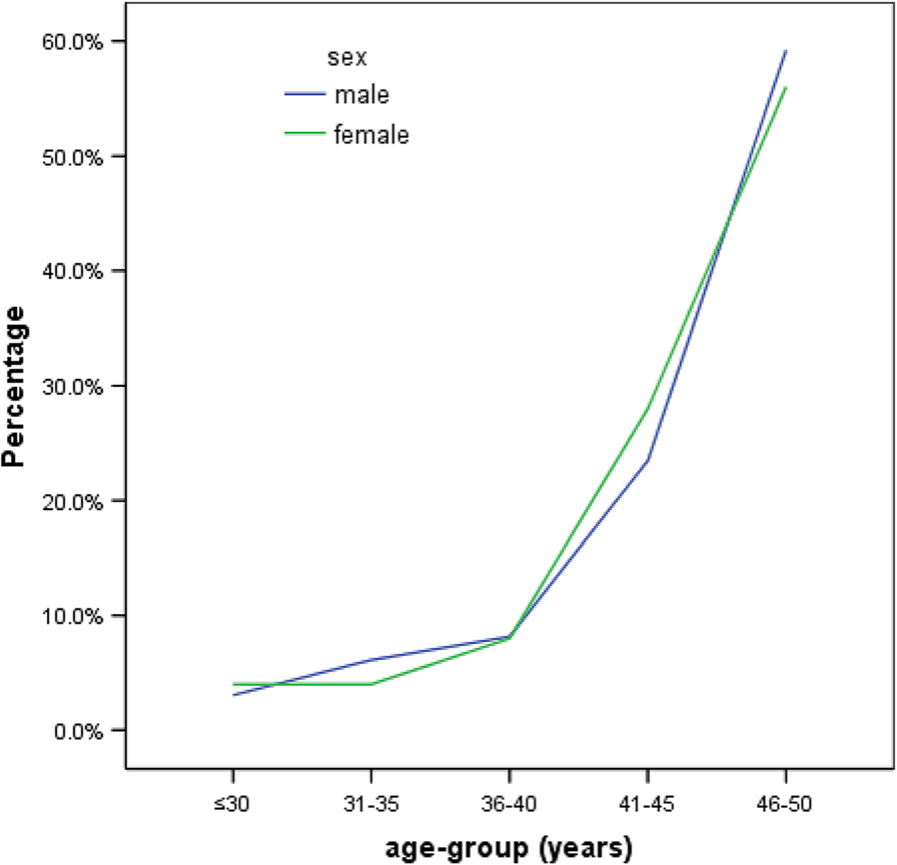
Ischemic infarctions stratified by sex and age groups

Our data pool was primarily comprised of males (male: 98; female: 25; male/female ratio: 3.92/1), shown in Table 1. The mean age of the study population was 44.50 ± 5.40 years (range: 25–50 years). Patients were admitted for 16.01 ± 4.82 days (range: 4–35 days). Patients were divided into two groups according to age-group: ≤45 and 46–50 years, in which 51 (41.46 %) fell under the former group and 72 (58.54 %) fell under the latter. Hypertension was the most common risk factor, 76.5 % in males and 58.3 % in females; *P* < 0.05. There were a significant number of male smokers (67.3 vs 4.0 %; *P* < 0.001). Mitral valve disease was more prevalent in female patients (12.0 vs 1.0 %; *P* < 0.05). Seventy-two patients (58.5 %) were in the 46–50 years age-group, 30 patients (24.4 %) in the 41–45 years age-group, 10 patients (8 %) in the 36–40 years age-group, 7 patients (5.7 %) in the 31–35 years age-group and 4 patients (3.3 %) were in the ≤ 30 years age group. Similar increase in stroke frequency with age was observed in both sexes (Fig. 1).

**Table 1:** Demography with risk factors and clinical features of young patients with ischemic stroke

	Male (*n* = 98)	Female (*n* = 25)	*P*-value	Patients ≤45 years (*n* = 51)	Patients 46–50 years (*n* = 72)	*P*-value
Age (years)	44.47 ± 5.48	44.60 ± 5.19	0.915	39.81 ± 5.01	48.16 ± 1.35	a
Smoking	66 (67.3 %)	1 (4.0 %)	<0.001^b^	25 (46.6 %)	41 (57.0 %)	0.385
Hypertension	75 (76.5 %)	14 (58.3 %)	0.040^c^	34 (62.1 %)	52 (70.9 %)	0.508
Dyslipidemia	57 (58.2 %)	11 (44 %)	0.204	32 (56.9 %)	36 (49.4 %)	0.161
Diabetes	36 (36.7 %)	5 (20 %)	0.364	14 (25.9 %)	25 (36.7 %)	0.393
Atrial Fibrillation	2 (2.0 %)	0 (0 %)	a	1 (1.7 %)	1 (1.3 %)	a
Mitral valve disease	1 (1.0 %)	3 (12.0 %)	0.026^c^	4 (7.8 %)	0 (0 %)	a
Prior TIA/Stroke	13 (13.3 %)	2 (6.7 %)	0.734	5 (8.6 %)	10 (13.9 %)	0.495
Essen stroke risk score	2.02 ± 0.92	0.84 ± 0.75	<0.001	1.59 ± 0.962	1.93 ± 1.02	0.066
NIHSS at admission	3.53 ± 2.90	3.64 ± 3.48	0.872	3.96 ± 3.13	3.23 ± 2.91	0.183
NIHSS at discharge	1.97 ± 2.26	1.76 ± 2.22	0.679	2.24 ± 2.40	1.68 ± 2.09	0.171
mRS at admission	1.93 ± 1.19	1.76 ± 1.39	0.544	2.19 ± 1.33	1.67 ± 1.11	0.020^c^
mRS at discharge	1.23 ± 1.12	1.20 ± 1.26	0.893	1.54 ± 1.34	0.99 ± 0.89	0.011^c^
Barthel index at admission	83.52 ± 20.157	84.40 ± 23.06	0.850	78.15 ± 24.28	88.04 ± 16.25	0.012^c^
Barthel index at discharge	93.47 ± 13.67	93.00 ± 14.57	0.880	89.91 ± 17.00	96.09 ± 9.96	0.020^c^

Data are expressed as n (%) or mean ± standard deviation

*mRS* modified Rankin Scale, *NIHSS* The National Institute of Health Stroke Scale, *TIA* transient ischemic attack

a: *p* value not applicable; ^b^ *P* < 0.001; ^c^ *P* < 0.05

The NIHSS of patients did not differ among males and females (*P* = 0.872 at admission and *P* = 0.679 at discharge) and age -groups (*P* = 0.183 at admission and *P* = 0.171 at discharge), shown in Table 1. The MRS and Barthel index at admission and discharge were also similar in males and females. The MRS of patients in the ≤ 45 years and 46–50 years age-groups at admission was 2.19 ± 1.33 and 1.67 ± 1.11 respectively (*P* = 0.020); mRS at discharge was 1.54 ± 1.34 and 0.99 ± 0.89 respectively (*P* = 0.011). Similarly the Barthel index of patients in the ≤ 45 years and 46–50 years age-groups at admission was 78.15 ± 24.28 and 88.04 ± 16.25 respectively (*P* = 0.012); the Barthel index at discharge was 89.91 ± 17.00 and 96.09 ± 9.96 respectively (*P* = 0.020).

**Fig. 2:**
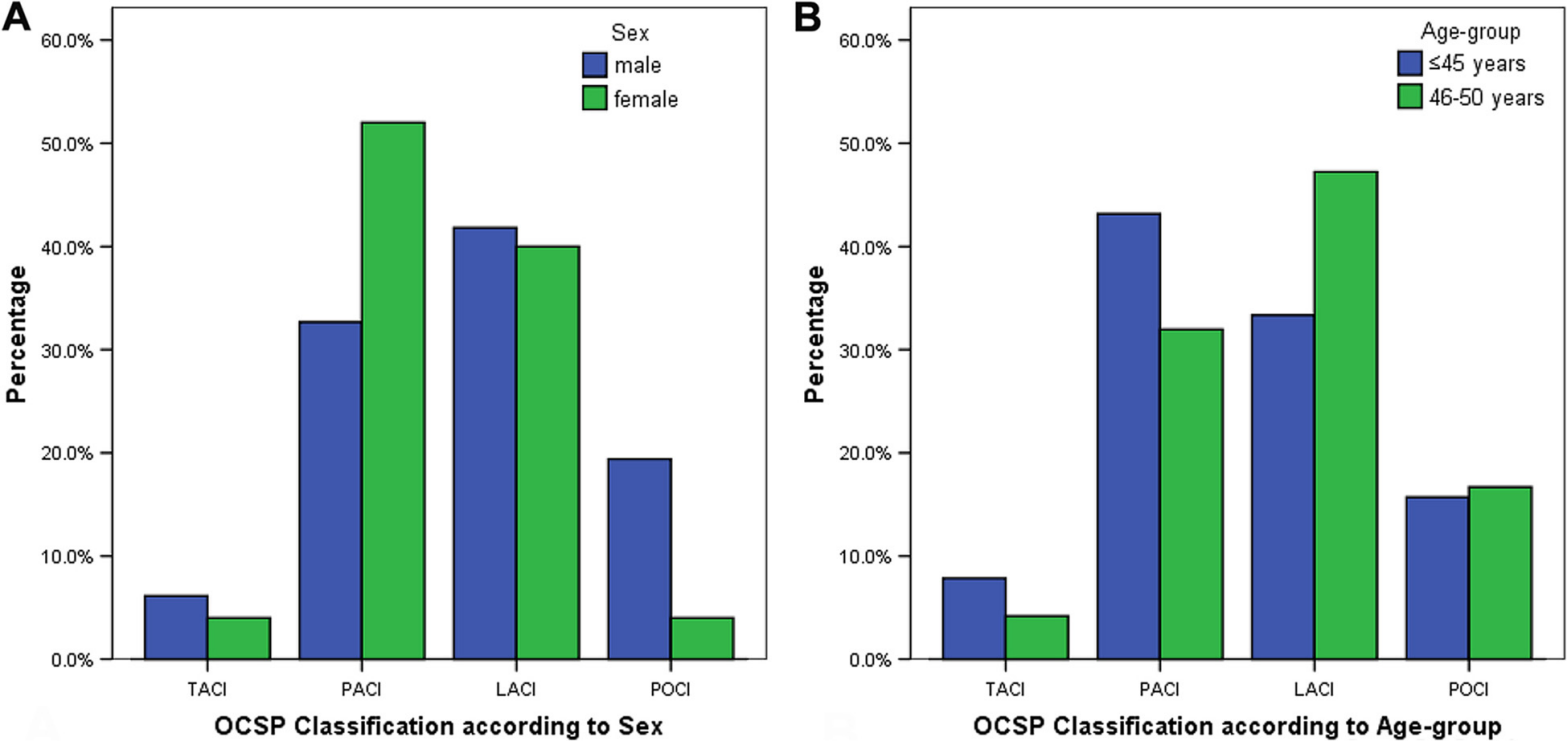
OCSP findings according to **a** sex and **b** age-group. No significant differences were observed while comparing young patients with ischemic stroke in terms of sex (male and female) and age-group (≤45 years and 46–50 years). LACI: Lacunar infarction; OCSP: Oxfordshire Community Stroke Project; PACI: Partial anterior circulation infarction; POCI: Posterior circulation infarction; TACI: Total anterior circulation infarction

**Fig. 3:**
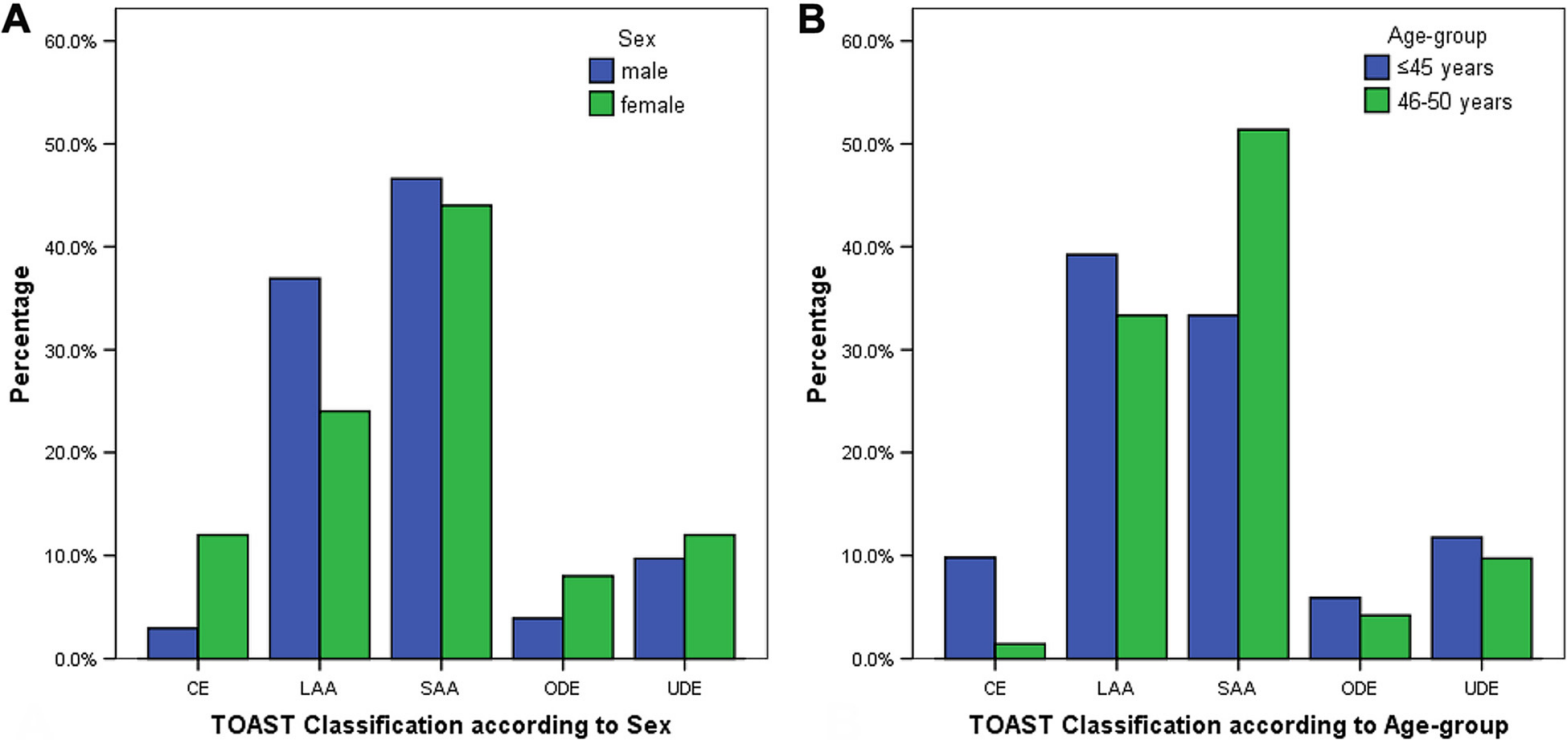
TOAST findings according to **a** sex and **b** age-group. SAA was found to be more prevalent in patients of the age group 46–50 years than in patients ≤45 years (*P* value < 0.05). No significant differences were observed while comparing young patients with ischemic stroke in terms of sex (male and female). CE: Cardioemblolism; LAA: Large artery atherosclerosis; ODE: Other determined etiology; SAA: Small artery atherosclerosis; TOAST: Trial of Org 10172 in Acute Stroke Treatment; UDE: Undetermined etiology

According to OCSP classification, 41.5 % of our patients had LACI, 36.6 % had PACI, 16.2 % had POCI and 5.7 % had TACI. There were no statistical differences in OCSP subtypes among the different sex and age-groups. However, PACI was predominant in female patients (52.0 vs 32.7 %; *P* = 0.073) and POCI in male (19.8 vs 4 %; *P* = 0.073), shown in Fig. 2. According to TOAST classification, SAA was the most common (43.9 %) in our patients with higher frequency in the age-group 46–50 years (51.4 vs 33.3 %; *P* < 0.05), shown in Fig. 3. Similarly, LAA was found in 44 patients (35.8 %), cardioembolism in 6 patients (4.9 %), ODE in 6 patients (4.9 %) and UDE in 13 patients (10.6 %). No significant differences in TOAST subtypes were found between male and female patients. ODE subtype included 6 patients (4.9 %): arterial dissection (male:1), migraine (female:1), tumor emboli (female:1), neurosyphilis (male:2) and vasculitis (male:1).

The DWI findings showed basal ganglia infarction was more frequent in young ischemic stroke (22.8 %), followed by thalamus (13.0 %) and corona radiata (8.1 %) (Table 2). Distribution of ischemic infarctions was not significantly different among the different sex and age-groups. Thirty-eight patients (30.9 %) were found to have MBI, out of which, 1 case had bilateral hemisphere infarction, 2 cases had bilateral cerebellar infarction and 2 cases had infarction of both brainstem and cerebral hemisphere. LAA was the cause of MBI in 19 patients (50 %), cardioembolism in 6 patients (15.8 %), ODE in 5 patients (13.2 %) and UDE in 8 patients (21 %). We also had one case of familial autosomal dominant polycystic kidney disease (ADPKD). Thirteen patients (12.2 %) had isolated brainstem infarctions: 8 pontine (6.5 %), and 5 medullary (4.1 %). No midbrain infarction was reported in our patients. Six patients (4.9 %) had unilateral cerebellar infarction.

**Table 2:** Distribution of ischemic infarctions according to sex and age-group

Distribution of ischemic infarctions	Total frequency n (%)	Sex			Age-group		
		Male n (%)	Female n (%)	*P*-value	≤45 years n (%)	46–50 year n (%)	*P*-value
Basal ganglia	28 (22.8)	21 (21.4)	7 (28.0)	0.484	10 (19.6)	18 (25.0)	0.482
Frontal lobe	3 (2.4)	2 (2.0)	1 (4.0)	*0.497	1 (2.0)	2 (2.8)	a
Occipital lobe	2 (1.6)	2 (2.0)	0 (0.0)	a	0 (0.0)	2 (2.8)	a
Parietal lobe	5 (4.1)	4 (4.1)	1 (4.0)	a	2 (3.9)	3 (4.2)	a
Thalamus	16 (13.0)	12 (12.2)	4 (16.0)	*0.739	5 (9.8)	11 (15.3)	0.374
Corona radiate	10 (8.1)	7 (7.1)	3 (12.0)	*0.423	6 (11.8)	4 (5.6)	*0.316
Cerebellum	6 (4.9)	6 (6.1)	0 (0.0)	a	1 (2.0)	5 (6.9)	*0.399
Pons	8 (6.5)	7 (7.1)	1 (4.0)	a	1 (2.0)	7 (9.7)	*0.071
Medulla	5 (4.1)	4 (4.1)	1 (4.0)	a	4 (7.8)	1 (1.4)	0.159
Multiple brain infarction	38 (30.9)	31 (31.6)	7 (28)	0.726	20 (39.2)	18 (25.0)	0.093
Periventricle	2 (1.6)	2 (2.0)	0 (0.0)	a	1 (2.0)	1 (1.4)	a
Total	123 (100)	98 (100)	25 (100)		51 (100)	72 (100)	

Data are expressed as n(%)

a: *p* value not applicable

*Fisher’s Exact Test

Duplex sonography reported 68 cases (55.3 %) of atherosclerosis, out of which 22 cases (34.4 %) had a plaque. While correlating the lesion site and involved artery, intracranial stenosis was more frequent (38.2 %) while extracranial stenosis was observed only in 13 % of the cases. MCA was the most common artery to be stenosed in young patients with ischemic stroke (27.6 %) with no significant difference in frequency among different sex and age-groups (Table 3). Fifty cases had their stenosis located in anterior circulation whereas only 17 cases have stenosis in the posterior circulation (*P* < 0.001). Involvement of multiple arterial stenosis was observed in 53 (43.1 %) patients. Frequencies of multiple arterial stenosis in the ≤45 years age group and the 46–50 years old age group were 17 cases (33.3 %) and 36 cases (50 %) respectively; 45 cases (45.9 %) and 8 cases (32.0 %) were male and female respectively.

**Table 3:** Distribution of intra- and extracranial artery stenosis according to sex and age-group

Stenotic artery	Total frequency n (%)	Gender			Age-group		
		Male n (%)	Female n (%)	*P*-value	≤45 years n (%)	46–50 years n (%)	*P*-value
ICA	7 (5.7)	5 (5.1)	2 (8.0)	*0.624	2 (3.9)	5 (6.9)	0.698
VA	5 (4.1)	4 (4.1)	1 (4.0)	a	4 (7.8)	1 (1.4)	*0.158
MCA	34 (27.6)	27 (27.6)	7 (28.0)	0.895	17 (33.3)	17 (23.6)	0.225
ACA	5 (4.1)	3 (3.1)	2 (8.0)	*0.257	3 (5.9)	2 (2.8)	0.648
PCA	5 (4.1)	3 (3.1)	2 (8.0)	*0.257	2 (3.9)	3 (4.2)	a
PICA	3 (2.4)	3 (3.1)	0 (0.0)	0	1 (2.0)	2 (2.8)	a
ICA and VA	4 (3.3)	3 (3.1)	1 (4.0)	a	2 (3.9)	2 (2.8)	a
MAS	53 (43.1)	45 (45.9)	8 (32.0)	0.241	17 (33.3)	36 (50)	0062
Total	116 (100)	93 (100)	23 (100)		48 (100)	68 (100)	

Data are expressed as n(%)

*ICA*, internal carotid artery, *VA* vertebral artery, *MCA* middle cerebral artery, *ACA* anterior cerebral artery, *PCA* posterior cerebral artery, *PICA*, posterior inferior cerebellar artery, *MAS* multiple artery stenosis

a: *p* value not applicable

*Fisher’s Exact Test

## Discussion

The occurrence of stroke in a relatively young population is more frequent among males as observed in our study. However, some studies have reported equal numbers of male and female patients [3, 13]. Hypertension and smoking were highly prevalent among young male patients in our study. A similar result was reported from a Chinese study of risk factors in a large series of young stroke patients [2]. In contrast to our study, western studies have shown a higher prevalence of smokers among young female patients with stroke [24, 25]. Even Essen stroke risk score was significantly high among male patients. Female patients were also found to have lower tendency to other risk factors like dyslipidemia, diabetes and atrial fibrillation. Furthermore, estrogen in females may have been protective against ischemic stroke as it has positive effects upon cerebral circulation [26]. This may explain low frequency of stroke among Chinese females. However, few studies have reported female stroke cases to be more frequent than males below the age of 30 [3, 27] and to be less frequent during the premenopausal and perimenopausal periods [28]. Previous research suggests the use of oral contraceptive pills, pregnancy and higher incidence of migraine among females can also result in high frequency of stroke in this age-group [29]. We only had one case of female migraine stroke and a similar increase in number of male and female patients was seen with age (Fig. 1).

The NIHSS score of our patients showed that there was no difference in severity of neurological deficits among different sex and age-groups (Table 1). Comparison of MRS and Barthel index also showed no difference in the functional disability was observed between males and females, however, significant functional disability was observed in the younger age-group patients both during admission and discharge. Functional disability in the younger age group might be due to the lower frequency of SAA and higher CE.

According to OCSP classification, lacunar Infarction was the most frequent in our study, followed by PACI. PACI was common in young stroke in various western studies such as those in Italy, Switzerland and the United Kingdom [30–32]. A higher frequency of hypertension and dyslipidemia in this population of young patients might be the reason for predominance of lacunar Infarction. Our study showed PACI was predominant in females, while POCI in males. New England medical center posterior circulation registry also reported that POCI was found to be more associated with the male sex [33]. High exposure to hypertension and smoking, and lower capacity for autoregulation of posterior circulation in males might be the reason for the POCI dominance [34, 35].

According to TOAST classification, predominance of SAA was seen in both male and female patients. Predominance of SAA was reported in the 46–50 years age group patients, which could be due to their long-term exposure to risk factors like hypertension, dyslipidemia and diabetes. Similarly, higher prevalence of SAA was reported from Asian countries like, Malaysia [36], Korea [37] and Taiwan [38], whereas Western [39] and a few Asian [40] studies showed LAA as the most common cause of young stroke. Various Asian and European young stroke studies reported cardioembolism in a range of about 10–25 % [7, 13, 31, 37, 38]. The few cardioembolic cases in our study may be due to the high mean age of study population and less prevalence of risk factors like atrial fibrillation and valvular heart diseases. Atrial fibrillation was reported only in male patients and mitral valve disease was more prevalent in females. Since trans-esophageal echocardiography was not done in our patients, we might have missed some other cardiac pathologies. Western studies usually report high frequency of ODE, arterial dissection being most common [7, 13, 31]. However, we observed only a few cases of ODE with a single case of arterial dissection in our study group.

Infarction in the basal ganglia was found to be most frequent in our study, followed by infarction of the thalamus and corona radiata. The high frequency of SAA in our study might be the reason for higher incidence of basal ganglia and thalamus infarction [41]. One of our young stroke cases was a familial ADPKD who had lacunar infarction located in the basal ganglia with normal vessel status in imaging analysis. ADPKD with ischemic stroke is a rare condition, and only a few cases have been reported previously [42]. But the association between ischemic stroke and ADPKD could not be confirmed with our investigations. There was a predominance of anterior circulation stroke found in both sexes, a similar result was reported in young stroke studies in Norway [27] and Switzerland [31]. Brainstem and cerebellar infarctions usually comprise around 20 % of total stroke [43] which is in accordance with the present study (Table 2).

MBI is a common finding in young stroke studies. A Finnish study of 185 young stroke patients found MBI in 33.8 % of the study group [44]. Among the MBI cases, 144 had single vascular territory and 41 had multiple vascular territory involvement. Similar results were reported in a Korean (28.9 %) [45] and a Swiss study (30.2 %) [46]. Usually MBI in young patients suggests cardioembolism, but LAA was the most common finding among our MBI patients, followed by UDE, cardioembolism and ODE. In the LAA subtype, MBI may be due to hemodynamic compromise resulting from stenotic vessels. Some of the patients had infarctions in the watershed zone suggesting that the coexistence of hypoperfusion with LAA could have led to MBI.

Similar to our study, predominance of intracranial over extracranial stenosis have been previously reported [47]. Intracranial stenosis is more predominant in East-Asian and African American people, while extracranial stenosis in whites and West-Asian people [14, 48]. A recent study suggested that higher levels of antioxidants in intracranial vessels at a young age may lead to lower occurrence of ICA stenosis [49]. However, the contrasting results among Chinese stroke studies are not clear and require further investigation.

Anterior circulation stenosis of cerebral vessels was significantly higher in our study. A recent comparative study between stroke patients of ≤45 years and >45 years also showed the higher incidence of cerebral stenosis in anterior circulation and higher in frequency among young females [8]. MCA is usually the most common artery vulnerable to stenosis in both young and old stroke patients [7, 11, 50], a similar finding was observed in our study. Though we had several patients with brainstem and cerebellar infarctions, involvement of basilar artery was not observed in our study. One reason might be that only head MRA was insufficient to assess the basilar artery and cervical MRA was performed only in a few patients. Involvement of multiple arterial stenosis was higher in 46–50 years age-group patients. An age related atherosclerosis with long-term risk factors like hypertension, smoking, dyslipidemia and diabetes might be the reason for higher frequency of multiple arterial stenosis.

### Limitations

This is a single centre study and we were unable to recruit the desired number of patients. Female stroke patients were comparatively small in number. Since this is a retrospective study, we had to rely on the patients’ reports stored in computer database of our hospital. Complete reports of imaging studies were not obtained from some patients and no graduation of stenosis was reported. Further, patients’ reports lacked information about risk factors like genetics, oxidative stress markers, abdominal obesity, physical inactivity, amount of alcohol consumption and intake of fruits and vegetables. No contrast enhanced MRA were used and 3D MRA TOF images might have suffered from blood-flow artifacts that mimic stenosis. Since trans-esophageal echocardiography was not done in our patients, we might have missed some cardiac pathology.

## Conclusions

Intracranial stenosis is more common in young stroke, with the MCA being highly vulnerable to stenosis. Higher frequency of hypertension and dyslipidemia in young patients resulted in predominance of lacunar infarction. Further, Chinese male patients are highly exposed to smoking and hypertension. Such risk factors should not be overlooked and lifestyle changes, regular health checkup and pharmacological interventions should be encouraged for primary and secondary prevention of young stroke. Further studies are needed to verify the high frequency of ICA stenosis in Chinese young adults.

### Ethics

The study protocol was approved by the Ethics Committee of East Hospital, affiliated to Tongji University School of Medicine.

### Consent

Written informed consent was obtained from all patients.

### Availability of supporting data

The dataset supporting the results of this article are included within the article.

## Abbreviations

CT: computed tomography
DWI: diffusion weighted imaging
LAA: large artery atherosclerosis
MBI: multiple brain infarcts
MRA: magnetic resonance angiography
mRS: modified Rankin Scale
NIHSS: National Institutes of Health Stroke Scale
OCSP: Oxfordshire Community Stroke Project
ODE: other determined etiology
PACI: partial anterior circulation infarction
POCI: posterior circulation infarction
SAA: small artery atherosclerosis
TACI: total anterior circulation infarction
TOAST: Trial of Org 10172 in Acute Stroke Treatment
UDE: undetermined etiology
